# High-Rate Bioelectrochemical Anaerobic Digester for Biomethane Production from Food Waste

**DOI:** 10.3390/bioengineering13010031

**Published:** 2025-12-27

**Authors:** Virender Singh, Abid Hussain, Banu Örmeci, Julien Pauzé-Foixet, Emmanuel Nwanebu, Hongbo Li, Boris Tartakovsky

**Affiliations:** 1 Department of Civil and Environmental Engineering, Carleton University, 1125 Colonel by Drive, Ottawa, ON K1S 5B6, Canada; 2 National Research Council of Canada, 6100 Royalmount Ave, Montreal, QC H4P 2R2, Canada

**Keywords:** bioelectrochemical digester, food waste, microbial electrolysis, waste management

## Abstract

This study investigated methane (CH_4_) production in a bioelectrochemically enhanced anaerobic digester (BEAD) equipped with a pair of 3-dimensional flow-through electrodes made of conductive polypropylene biorings. The performance of the BEAD reactor was compared to that of a similarly sized Anaerobic Upflow Sludge Bed (UASB) reactor. The reactors were operated at a temperature of 22 ± 1 °C using food waste (FW) leachate fed at organic loading rates of 3–8 g (L_R_ d)^−1^ or at a temperature of 35 ± 1 °C using the liquid fraction of FW separated using a screw press. With both tested feedstocks, the BEAD reactor demonstrated up to 30% higher CH_4_ yield, reaching 0.35–0.38 L g^−1^ (COD consumed), compared to the UASB reactor. Additionally, reactor stability under organic overload conditions improved, with the difference more pronounced at organic loads above 6 g (L_R_ d)^−1^. Energy consumption for bioelectrochemical CH_4_ production was estimated at 5.1–12.4 Wh L^−1^ (of CH_4_ produced), which is significantly below the energy consumption for electrochemical H_2_-based methanation. Overall, BEAD increases methane production and improves process stability, offering a novel sustainable solution for waste management.

## 1. Introduction

Although anaerobic digestion (AD) is an excellent technology for converting waste organics into biogas, only a small fraction of biodegradable organics is actually used for biogas production [1]. According to the International Energy Agency’s 2020 report, biomethane production in 2018 amounted to approximately 35 million tons of oil equivalent (Mtoe), accounting for only 6% of the global biogas production potential of 570 Mtoe [2]. Common challenges hindering widespread AD adoption for methane production from organic wastes include the high transportation costs of organic wastes to AD facilities, the substantial costs of constructing anaerobic reactors, the relatively low volumetric rates of biogas production often limited by hydrolysis, and the susceptibility of the AD process to various external uncontrollable factors [3,4]. Municipal anaerobic digesters typically operate with hydraulic retention times exceeding 20 days [5,6,7], whereas high-rate anaerobic systems like Upflow Anaerobic Sludge Bed (UASB) reactors face limitations in processing feedstocks with high solid contents and require highly skilled operators for efficient operation [8]. Additionally, fluctuations in feedstock quantity and quality often result in organic overloads, leading to acidification events that can severely impact methanogenic populations [9,10]. Recovering from such disturbances can be extremely long, sometimes necessitating re-inoculation to fully restore reactor performance. Moreover, successful AD operation requires relatively high temperatures (35–38 °C), which pose challenges in colder regions where energy requirements for reactor heating may nearly equal energy production [11].

Recent advances in the development of the bioelectrochemically enhanced anaerobic digestion (BEAD) process offer a promising approach to resolve some of the limitations associated with conventional anaerobic reactors. A BEAD reactor is based on the microbial electrolysis cell (MEC) concept and integrates conventional pathways for organic matter hydrolysis, fermentation, and methane production with bioelectrochemical pathways involving electroactive microorganisms capable of direct or indirect electron exchange with electrodes [12,13,14,15,16,17,18,19,20]. Accordingly, it includes at least one pair of flow-through electrodes (anode and cathode). In this configuration, anaerobic exoelectrogenic microorganisms, such as *Geobacter* spp., oxidize organic matter using the anode as a terminal electron acceptor. To overcome thermodynamic limitations, electron flow to the cathode is assisted by a power supply operated below the onset of water electrolysis [13,15,16,17,21,22,23,24,25]. At the cathode, H_2_ produced electrochemically or with the assistance of electroactive microorganisms is utilized for CH_4_ production. Recent studies have identified several pathways leading to CH_4_ production, including direct and indirect electron transfer mechanisms, as well as direct electromethanogenesis from CO_2_ [17,18,21,26,27]. This combination of pathways leads to increased CH_4_ yield in bioelectrochemical systems, which can be attributed to the higher affinity of anodophilic electroactive microorganisms for acetate and other acidified substrates [16,28,29,30] and enhanced electron exchange between the members of syntrophic microbial consortium [31]. In addition to increased biogas production, improved reactor stability under varying operational conditions and stable performance at lower mesophilic temperatures have been reported [15,20].

The slow hydrolysis of complex organic substrates, such as food waste, is the main factor limiting the rate of CH_4_ production and COD removal in anaerobic reactors [32,33,34,35]. To resolve this limitation, a two-phase process typically includes a pre-treatment step in a bioreactor operated under conditions optimized for organic substrate hydrolysis and fermentation [36]. This pre-treatment step can be accomplished in a dry fermenter such as a Leach Bed Reactor (LBR) [37]. LBRs are attractive as a low cost and low energy consumption approach for pretreatment. Nevertheless, this pretreatment system faces limitations such as clogging of the waste-holding chamber and prolonged fermentation times of up to 14–18 days. A recently developed solid-state submerged fermenter (3SF) offers solutions for overcoming these limitations [38]. This new reactor design allows for a higher volumetric loading rate as well as shorter fermentation times of 8–10 days. Furthermore, new pre-treatment methods such as solid–liquid separation with a screw press [39], hydrothermal pretreatment [33,40,41,42] and coupled alkali-microwave-H_2_O_2_ oxidation pretreatment [43] have emerged.

This proof-of-concept study aimed to evaluate the performance of a UASB-style BEAD reactor with an upflow configuration of three-dimensional flow-through electrodes. To enable a direct performance comparison, a conventional UASB (Control) reactor was operated simultaneously with the BEAD reactor. Since the effective operation of high-rate anaerobic reactors based on the UASB design requires feedstock with low solid contents, food waste was pretreated in all experiments. The broad applicability of the proposed approach to various types of organic waste and solids pretreatment methods was demonstrated using two distinct feedstocks: leachate from a 3SF LBR reactor fed with synthetic food waste and the liquid fraction of food waste collected from a local food bank and separated using a screw press, i.e., a two-phase anaerobic digestion process was adopted for the experiments. To ensure the robustness and broad applicability of the proposed approach, experiments were conducted by two independent research groups at different geographical locations using different feedstocks and two different mesophilic temperatures.

## 2. Materials and Methods

### 2.1. Inoculum and Feedstock Characteristics

All reactors used for CH_4_ production from FW leachate were inoculated with homogenized granular anaerobic sludge treating agriculture waste (Lassonde Inc., Rougemont, QC, Canada) with an average VSS content of 40–45 g L^−1^. Reactors used for treating screw press separated (SPS) liquid were inoculated with the same inoculum; however, the anaerobic sludge was not homogenized.

Food waste leachate was obtained in a 3SF LBR operated on simulated FW. This simulated FW was composed of carrots (40%), potatoes (35%), bread (15%), and pet food (10%). Leachate was collected at the end of each LBR batch (approximately 8–10 days in duration). The total suspended solids (TSS) and volatile suspended solids (VSS) of the FW leachate were 4.9% ± 0.05% and 2.7% ± 0.03%. For initial tests, the leachate was centrifuged at 10,000 rpm for 5 min (THERMO SCIENTIFIC, Waltham, MA, USA, Sorvall, Legend RT+ Centrifuge, rotor diameter 19 cm) to remove all remaining solids, while non-centrifuged leachate was used for subsequent tests.

For experiments involving screw press separated (SPS) liquid, food waste was procured from a food bank (Moisson Montreal) located in Montreal, QC, Canada. Solid–liquid separation was achieved with a CP-4 screw press (Vincent Corporation, Tampa, FL, USA). The screw press was operated at 20 rpm with an applied pressure of 60 psig. The liquid fraction was collected and stored at 4 °C until further use. Detailed description of the screw press operation conditions can be found elsewhere [39].

### 2.2. Reactor Design and Location

Experiments were conducted at two different laboratories located at Carleton university (Ottawa, ON, Canada) and at the National Research Council of Canada (NRC, Montreal, QC, Canada).

At each location, all experiments were conducted using two simultaneously operated cylindrical glass reactors (BEAD and Control). Since the experiments were conducted independently at two different laboratories, slightly different reactor dimensions were employed. At Carleton university (Ottawa), each reactor had a height of 435 mm and a diameter of 54 mm, resulting in a total volume of 1 L and a working (liquid) volume of 0.65 L. At the NRC (Montreal), the reactors had the same design and internal diameter, however each reactor had a working liquid volume of 0.45 L.

In all experimental setups, reactors were equipped with external recirculation loops. An upflow velocity of 1–2 m h^−1^ was maintained using peristaltic pumps. Also, the reactors were equipped with temperature and pH probes. Heating elements wrapped around the external recirculation lines were used to maintain a preset temperature. Reactor pH was maintained at a preset level using a pH controller connected to a peristaltic pump, which added either 0.25 N HCl solution or 0.25 N NaOH solution into the external recirculation loop.

The BEAD reactor used a membrane-less, flow-through electrode configuration. Titanium current collectors (meshes) measuring 120 mm in height, 20 mm in width, and 2 mm in thickness, were used in both the anode and cathode compartments. The flow-through electrodes were composed of densely packed custom-made electrically conductive biorings (c-biorings) with each electrode compartment containing 30 c-biorings (Montreal) or 42 c-biorings (Ottawa). The c-biorings had a hollow cylinder shape with an approximate surface area of 11.4 cm^2^, an internal diameter of 15 mm, a wall thickness of 2 mm, a height of 12 mm and a weight of 1.7 g. These c-biorings were made of Polypropylene containing 12% carbon black (CB) and 2% carbon nanotubes (CNT), resulting in a conductivity of 200–250 Ohm (top to bottom).

**Figure 1:**
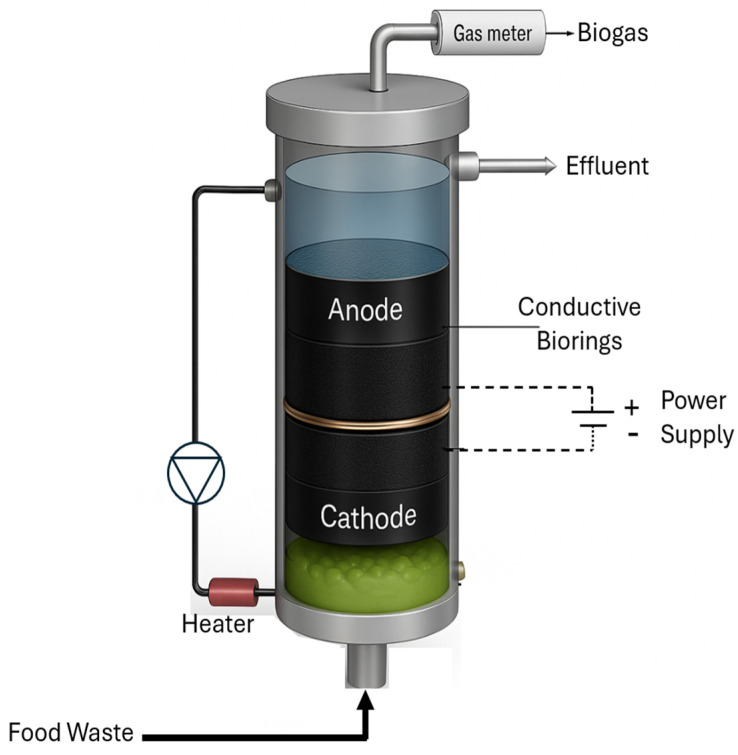
BEAD reactor diagram.

The electrode compartments were separated by a non-conductive 3 mm thick geotextile separator perforated with multiple 2–3 mm holes. The cathode compartment was positioned at the reactor bottom to enhance H_2_ solubilization. This configuration takes advantage of the longer time for H_2_ bubbles produced at the cathode to reach liquid surface. Figure 1 shows the schematic diagram of the BEAD reactor. The Control reactor had an identical design; however, it was filled with non-conductive Polypropylene biorings (Ottawa) and lacked power supply. The Control reactor in Montreal followed a standard UASB design and lacked microbial support.

### 2.3. Reactor Startup and Operating Conditions

For the startup of LBR leachate-fed reactors at Carleton university (Ottawa), both BEAD and Control reactors were inoculated with 550 mL of homogenized anaerobic sludge, followed by the addition of 100 mL of diluted centrifuged leachate with a COD concentration of 10 g L^−1^. During the first three weeks of the experiment, reactors operated in batch mode with 20 mL food waste (FW) leachate added daily to each reactor. Once biogas production and composition stabilized reaching more than 60% CH_4_ content, continuous mode of reactor operation started. A temperature of 22 ÷ 1 °C was maintained (room temperature).

The reactor startup procedure at the NRC laboratory (Montreal) was similar, but each reactor was inoculated with 150 mL of homogenized anaerobic sludge. At each location, various organic loading rates (OLRs) were applied to both reactors, as shown in Table 1. The corresponding hydraulic retention time (HRT) values varied between 3–12 days. A temperature of 22 ÷ 1 °C was maintained (room temperature).

In all experiments, each OLR was maintained until observing steady-state performance in terms of CH_4_ production (both reactors) and BEAD reactor current. Steady state was considered when observing less than a 10% variation in current and CH_4_ production for at least 3 consecutive days. Key performance parameters (CH_4_ production, yield, etc.) were compared using Student’s t-test, provided a sufficient number of measurements was available. In total, the BEAD and Control reactors were operated with food waste leachate for 68 days and 47 days in at Carleton University (Ottawa) and at the NRC laboratory (Montreal), respectively.

During the experiments involving reactor operation with screw press-separated (SPS) liquid feedstock in Montreal, the reactors were reinoculated and once steady state performance was observed operated for 26 days at a temperature of 35 °C. Similar to the experiments with FW leachate, tests using SPS liquid were conducted at several organic loads outlined in Table 1. Due to the significant presence of settleable fine solids in the SPS liquid, a settler with a hydraulic retention time of 4–5 days was utilized to partially remove these solids and facilitate hydrolysis and fermentation of the SPS liquid. Table 2 provides SPS liquid characterization at the settler exit.

At both locations, the feedstock was supplied to the reactors without removing trace oxygen (e.g., by flushing with N_2_), as multiple studies have demonstrated the positive impact of trace oxygen concentration on biogas production [44].

In all experiments involving FW leachate in Ottawa, Canada, reactors were operated at room temperature (22 ± 1 °C) and near-neutral pH (7.0 ± 0.2). FW leachate treatment tests were carried out in the BEAD reactor operated at a constant applied voltage of 1.4 V, which was maintained using a potentiostat (Metrohm, Mississauga, ON, Canada). Experiments utilizing screw press separated (SPS) liquid feedstock (Montreal, Canada) were also carried out at pH 7, but a temperature of 35 °C was maintained. Also, a lower applied voltage of 1.2 V was used throughout the SPS liquid fed BEAD reactor experiments.

### 2.4. Analytical Methods and Calculations

Chemical oxygen demand (COD) measurements were conducted using the Potassium Dichromate spectrophotometric method [45]. For the estimation of soluble COD (sCOD), samples were filtered using a 0.45 µm pore size filter membrane in a vacuum filter. The filtration step was omitted for total COD (tCOD) determination.

The influent and effluent concentrations of volatile fatty acids (VFAs) including acetate, propionate, and butyrate, were analyzed using HPLC (Thermo Scientific Ultimate 3000 series, Waltham, MA, USA). The mobile phase was composed of 100% Acetonitrile and 2.5 mM methanesulfonic acid and Acclaim^TM^ OA 5 µm, 4 × 150 mm column was used. The column temperature was maintained at 30 °C, with a fixed mobile phase flow rate of 1 mL min^−1^ and the detector’s absorption wavelength set at 210 nm.

Biogas production was quantified using gas flow meters (Milligas counter, Ritter North America Inc., Summerville, SC, USA). The biogas composition was measured using gas chromatography. Method details are provided elsewhere [46].

The COD Removal Efficiency (RE, %) was calculated by comparing the influent and effluent COD values, while CH_4_ yield calculations were based on the amount of CH_4_ produced relative to the amount of COD removed (L g^−1^).

Cathodic Coulombic Efficiency (*CE_c_*) was calculated as:

(1) $${CE}_{c}=\frac{mQ_{CH4}F}{\int_{o}^{T}idt}100$$

where *m* is the number of moles of electrons required to produce one mole of CH_4_ (m = 8); *F* is the Faraday constant (*F* = 96,485 C mol^−1^); *Q*_CH4_ is the flow of CH_4_, L d^−1^, *i* is the current (A); T is the total time in a day expressed in seconds (*T* = 86,400 s).

To estimate the contribution of bioelectroactive microorganisms to CH_4_ production in the BEAD reactor, CH_4_ production in the BEAD and Control reactors was compared under identical operating conditions as follows. The maximum theoretical CH_4_ production attributable to bioelectrochemical activity was estimated based on the electrical current measured in the BEAD reactor and the assumption of 100% CE_c_. Under this assumption, all electrons transferred through the cathode are used exclusively for the conversion of CO_2_ into CH_4_. Accordingly, Equation (1) can be rearranged:

(2) $$Q_{CH4}=\frac{\;\int_{o}^{T}idt\;}{\;m\;F}$$

Equation (2) provides the theoretical CH_4_ production, which could be produced if all measured current is used for CH_4_ formation. To compare the theoretical value with the actual increase in CH_4_ production, once CH_4_ production rates in the Control and BEAD reactors were determined under identical conditions, the difference between these two values, which represents actual CH_4_ production due to the applied voltage, was calculated.

Additionally, energy consumption for bioelectrochemical production of CH_4_ (Wh L^−1^) was calculated as:

(3) $$E_{CH4}=\frac{U\;I\;24}{V_{CH4}}$$

where *U* is the voltage (V), *I* is the current (A) and *V*_CH4_ is the volume of methane produced in 24 h (L).

## 3. Results and Discussion

This section presents the experimental results describing the evaluation of the proposed bioelectrochemical anaerobic digestion (BEAD) concept using centrifuged and non-centrifuged food waste leachate from the LBR reactor. To ensure the robustness and broad applicability of the proposed combined AD-MEC setup, the experiments were also conducted using screw press separated (SPS) food waste as a feedstock. Furthermore, the approach was tested at two distinctly different operating temperatures: 22 °C (food waste leachate feedstock tests) and 35 °C (SPS feedstock tests). By conducting the experiments simultaneously at two independent laboratories, further validation of the proposed approach was achieved.

### 3.1. Operation of BEAD and Control Reactors on Food Waste Leachate

As described in the Materials and Methods section, at both locations, reactor experiments were started by initially maintaining a low organic load within a range of 1.5–3 g (L_R_ d)^−1^ during the initial 3–4 weeks of operation (adaptation phase). In experiments utilizing FW leachate, reactors were operated on the centrifuged leachate before changing the feed to non-centrifuged leachate. Following the successful startup of each reactor, experiments were conducted with progressively increasing organic loads, as outlined in Table 1.

**Figure 2:**
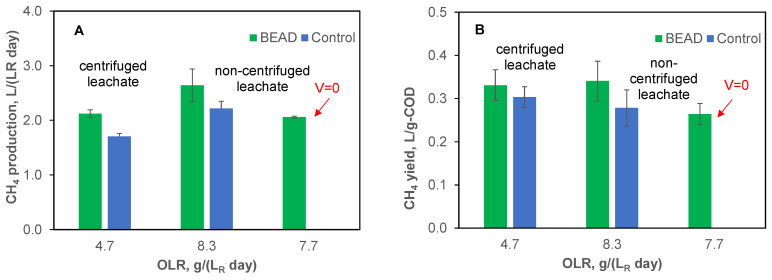
Specific rate of CH_4_ production (**A**) and CH_4_ yields (**B**) observed during BEAD and Control reactor operation at NRC (Montreal, reactor temperature 22 °C). Error bars represent the standard deviation.

During tests conducted in Montreal, following the adaptation phase, both BEAD and Control reactors were operated using centrifuged leachate at an organic load of 4.7 g (L_R_ d)^−1^ for a duration of 16 days (Test #1-1, Table 1). In the following experimental phase (Test #1-2), the feedstock was switched to the non-centrifuged leachate resulting in an OLR of 8.3 g (L_R_ d)^−1^. The resulting performance of the two reactors is compared in Figure 2, illustrating the average steady state volumetric rates of CH_4_ production (Figure 2A) and CH_4_ yields (Figure 2B).

As can be seen from this comparison, at both tested organic loads, CH_4_ production was higher in the BEAD reactor, with the difference becoming more pronounced at an OLR of 8.3 g (L_R_ d)^−1^. At this organic load, both CH_4_ production and yield were improved by 20–22%. At the same time, the biogas composition and COD removal efficiencies of the two reactors were comparable with values in a range of 79–80% and 92–95%, respectively. The observed high COD removal efficiency can be attributed to FW pretreatment in the LBR reactor, resulting in elevated concentrations of readily biodegradable short chain fatty acids, such as acetate.

Interestingly, reactor operation on the non-centrifuged leachate did not lead to a performance decline, suggesting that a significant part of leachate solids was also readily biodegradable. However, considering the relatively short duration of 18 days for this experiment, additional tests may be warranted to confirm stable long-term reactor performance and the absence of inert solid accumulation in the reactors.

To confirm the impact of microbial electrolysis on CH_4_ yield, in the following Test #1-3, conducted using non-centrifuged leachate, the BEAD reactor was operated at zero applied voltage (Figure 2). Due to variations in feedstock composition, the test was conducted at a slightly lower organic load of 7.7 g (L_R_ d)^−1^. Under these conditions, both CH_4_ production and yield decreased approaching the performance of the Control reactor, while the COD removal efficiency remained high (94–95%). These results suggest that bioelectrochemical conditions predominantly influenced CH_4_ yield. Previous studies have demonstrated the higher efficiency of direct electromethanogenesis as compared to CH_4_ production through such intermediates as H_2_ [26,47,48,49].

**Figure 3:**
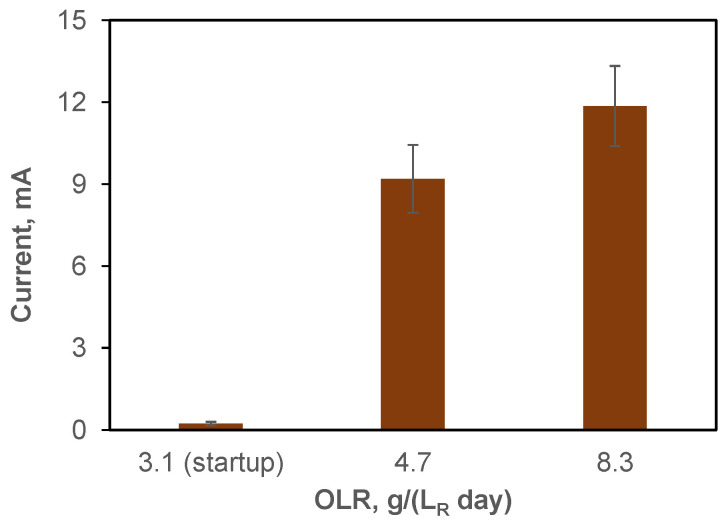
BEAD reactor current observed at the NRC laboratory (Montreal, reactor temperature 22 °C) during the startup phase and reactor operation at OLR values of 4.7 and 8.3 g (L_R_ d)^−1^. Error bars represent the standard deviation.

In addition to the observed differences in CH_4_ production, the proliferation of electroactive populations can also be inferred from the observed increase in current in the BEAD reactor over time (Figure 3). Notably, the current increased from 0.2 mA at reactor startup (day 1) to 12 ± 1.5 mA by the end of BEAD operation on non-centrifuged leachate (day 34). These values can be used to estimate the energy consumption for bioelectrochemical CH_4_ production by first calculating the difference between CH_4_ production in the BEAD and Control reactors and then dividing energy consumption by this value.

For BEAD reactor operation on centrifuged and non-centrifuged FW leachate, energy consumption estimates of 5.1 and 12.4 Wh $L_{CH4}^{-1}$, respectively, were calculated. Importantly, these values are significantly lower than the energy required for CH_4_ production using electrochemical H_2_. Assuming an energy consumption of 4.5–5.0 Wh $L_{CH4}^{-1}$ for electrochemical H_2_, based on average reported values for commercial electrolyzers [50] and considering the stoichiometry of CO_2_ to CH_4_ conversion, wherein a CO_2_/H_2_ molar ratio of 4 is required for hydrogenotrophic methanogenesis, an energy consumption of 18–20 Wh $L_{CH4}^{-1}$ is obtained. Therefore, the substantially lower energy consumption in the BEAD reactor can be interpreted as evidence of electromethanogenesis as one of the pathways leading to CH_4_ production at the BEAD reactor cathode. At the same time, energy consumption with respect to all CH_4_ produced in BEAD through a combination of conventional AD and bioelectrochemical pathways was only 0.1 Wh Wh $L_{CH4}^{-1}$.

**Figure 4:**
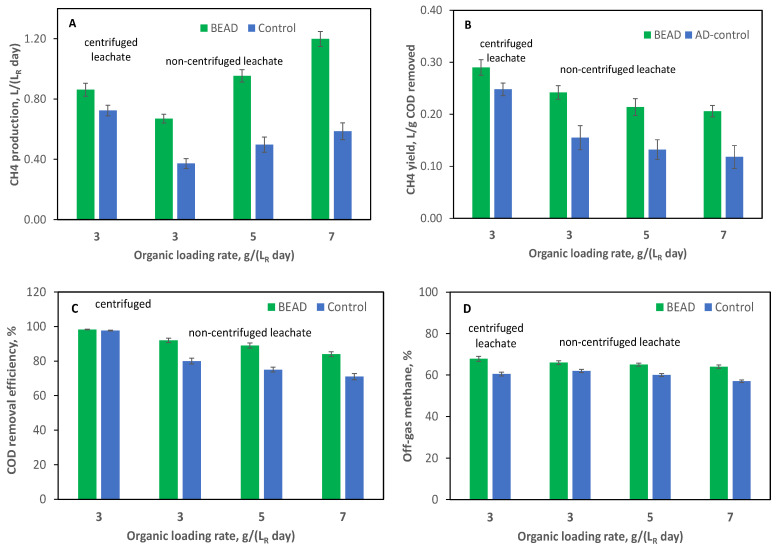
(**A**) CH_4_ production, (**B**) CH_4_ yield; (**C**) COD removal efficiency, and (**D**) CH_4_ percentage in the reactor off-gas observed during BEAD and Control reactors operation at Carleton university (Ottawa, reactor temperature 22 °C). Error bars represent the standard deviation.

To confirm the observed improvement in food leachate conversion to CH_4_, the operation of BEAD and Control reactors was repeated at the Carleton University laboratory (Ottawa). Results of this experiment are summarized in Figure 4. Similar to the experiments conducted at the NRC laboratory (Montreal), both reactors (BEAD and Control) underwent startup at a low organic load using centrifuged leachate (Table 1, Test #2-1). Figure 4 shows results observed during reactor operation at an OLR = 3 g (L_R_ d)^−1^_._ Which already showed improved CH_4_ production and yield in the BEAD reactor.

In the subsequent phase of the experiment, both reactors were fed with non-centrifuged leachate at progressively increasing OLRs (Tests #2-2 to #2-4). As can be seen from the results presented in Figure 4A, consistently higher biogas production was observed in the BEAD reactor as compared to the Control reactor at all tested organic loads with non-centrifuged leachate. Also, CH_4_ yields were significantly different between the two reactors at higher organic loads (Figure 4B). While CH_4_ yield declined in both reactors with the increase in organic load, this decline was substantially more pronounced in the Control reactor at the highest organic load, resulting in a 75% difference in CH_4_ yield between the reactors.

The two reactors demonstrated similar COD removal at an OLR of 3 g (L_R_ d)^−1^ during reactor operation on centrifuged leachate. However, BEAD exhibited higher removal at OLR values of 5 and 7 g (L_R_ d)^−1^ during reactor operation on non-centrifuged leachate (Figure 4C), with differences of up to 18%. Such difference was not observed in the previous set of experiments. It can be hypothesized that during a relatively brief previous experiment conducted at the NRC laboratory (Montreal), solids accumulated in the Control reactor, resulting in comparable COD removal efficiencies. Additionally, the CH_4_ percentage remained higher in the BEAD reactor off-gas throughout the experiment, with average values of 66% and 60% for the BEAD and Control reactors, respectively (Figure 4D).

At both locations, at the end of reactor experiments, c-biorings were removed from BEAD reactors and visually inspected. This inspection showed no observable signs of degradation after 68 days and 73 days of operation, at Ottawa and Montreal locations, respectively. Indeed, a comparison of different biofilm supports in anaerobic digestion demonstrated that polypropylene fiber and biorings media significantly increase CH_4_ production and COD removal [51,52]. Notably, an attempt to operate a UASB-style reactor using carbon felt at the Ottawa laboratory led to reactor clogging at high organic loading rates (unpublished results).

Overall, the results of experiments conducted at two different laboratories yielded qualitatively similar findings, suggesting that under bioelectrochemical conditions, conventional pathways for biomethane production were augmented by the activity of electroactive microorganisms, both at the anode and cathode. Moreover, the observed improvement in biogas yield agreed well with the recently published results of bioelectrochemically enhanced anaerobic digestion of industrial fishery wastewater, where a similar range of CH_4_ yield and current density was observed [28]. Additionally, methane production was observed to triple in a microbial electrolysis–anaerobic digestion (ME-AD) reactor treating waste activated sludge [22].

### 3.2. Biogas Production from SPS Liquid

To demonstrate the versatility of the BEAD reactor for treating various feedstocks, in addition to its operation on FW leachate, another set of experiments was conducted at the NRC laboratory (Montreal) using SPS liquid and a higher reactor temperature of 35 °C. Similar to previous tests, two rectors were simultaneously operated. However, to provide additional comparison, in this experiment, the Control reactor setup was changed to a more conventional UASB configuration by removing the non-conductive polypropylene biorings and inoculating both reactors with granular (non-homogenized) anaerobic sludge. The BEAD reactor design remained unchanged, although it was also inoculated with granular anaerobic sludge, as opposed to the homogenized sludge in the previous experiment.

Table 2 outlines key characteristics of the SPS feedstock utilized throughout this experiment. Notably, a settler was used to remove fine solids from the SPS liquid. Accordingly, the influent stream contained a relatively high concentration of short and medium chain fatty acids. Medium and long chain fatty acids are known to inhibit methanogenic microorganisms.

**Figure 5:**
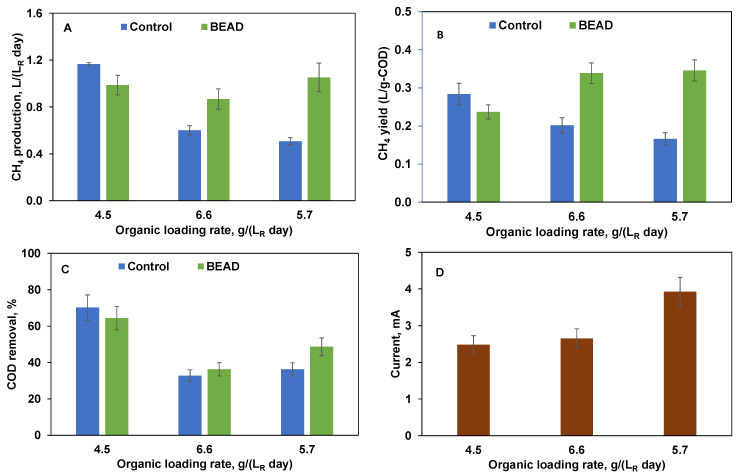
BEAD and Control reactor operation on screw press separated (SPS) food waste (Montreal, reactor temperature 35 °C) showing (**A**) CH_4_ production, (**B**) CH_4_ yield, (**C**) COD removal efficiency, and (**D**) BEAD current at three tested organic loading rates. Error bars represent the standard deviation of the measurements.

Figure 5 compares the performances of the two reactors at the three tested organic loads. At a relatively low organic load of 4.5 g (L_R_ day)^−1^, the two reactors demonstrated comparable performance, exhibiting similar average volumetric rates of CH_4_ production and CH_4_ yields, which is consistent with the results obtained in the two previous experiments using LBR leachate. Moreover, the COD removal efficiency and biogas composition were similar, with values ranging between 65–70% and 64–68%, respectively.

The differences between the two reactors became more apparent once the organic load was increased to 6.6 g (L_R_ day)^−1^. This organic load corresponded to organic overload conditions, with acetate, propionate, and butyrate concentrations increasing to 3700 mg L^−1^, 2020 mg L^−1^, 1200 mg L^−1^, respectively, and a total VFA concentration of 6920 mg L^−1^. The corresponding sCOD values were 9600 mg L^−1^. 14,090 mg L^−1^, respectively. Consequently, this organic overload led to a nearly 50% decrease in CH_4_ production in the Control reactor (Figure 5A). Also, CH_4_ yield in this reactor dropped below 0.2 L g^−1^ (Figure 5B), thereby confirming reactor overload.

At the same time, CH_4_ production in the BEAD reactor remained relatively unchanged (Figure 5B), although VFA concentrations were also high: 2340 mg L^−1^, 2420 mg L^−1^, 2150 mg L^−1^, respectively, resulting in a total VFA concentration of 6910 mg L^−1^. Remarkably, in spite of such high VFA levels, CH_4_ yield progressively increased towards the end of this phase, approaching 0.35 L g^−1^, which exceeded that at a load of 4.4 g (L_R_ day)^−1^ (Figure 5B).

In an attempt to restore the Control reactor performance, in the following experimental phase, the organic load was reduced to 5.7 g (L_R_ day)^−1^ for both reactors. Consequently, the volumetric rate of CH_4_ production improved in the BEAD reactor, while both CH_4_ production and yield further declined in the Control reactor. VFA analysis corroborated further increase of BEAD reactor capacity for COD removal showing a decrease in total VFA concentrations to 4860 mg L^−1^. Meanwhile total VFA concentration in the Control reactor increased to 7530 mg L^−1^ reflecting continuing organic overload.

**Figure 6:**
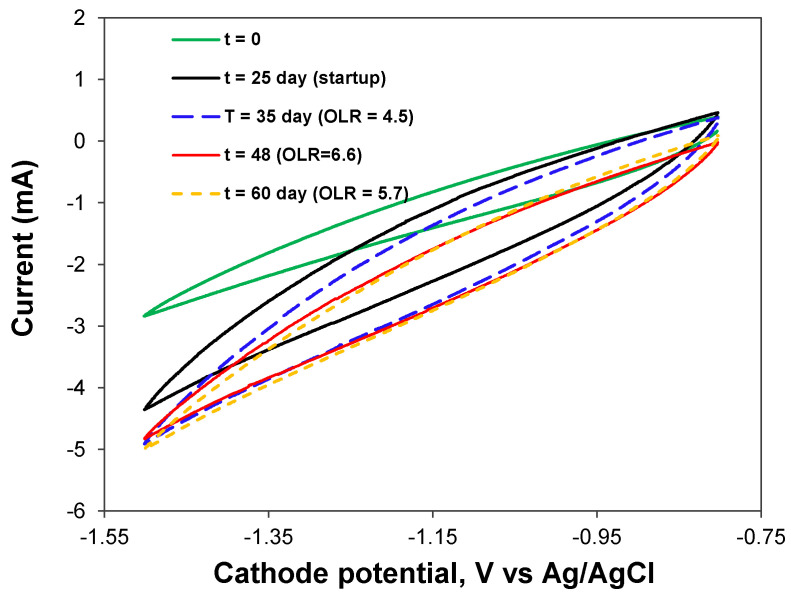
BEAD reactor cyclic voltammograms acquired at different phases of BEAD reactor operation.

Stable performance of the BEAD reactor with increasing activity of the electroactive bacteria was confirmed through current measurements (Figure 5D) and cyclic voltammetry (Figure 6). Both measurements indicated a progressive increase in current, indicative of an augmented population of electroactive bacteria. In addition, the broadening of the cyclic voltammograms (CV) relative to that at the experiment startup can be attributed to the growth of electroactive biofilm. Considering the absence of metal catalysts at both anode and cathode electrodes, it can be inferred that anodophilic and cathodophilic biofilms developed on the surface of conductive biorings, although electrochemical oxidation of organic materials at the anode cannot be completely excluded.

As discussed earlier, the improved CH_4_ production can be attributed to both direct and indirect (through acetate and hydrogen) pathways of CH_4_ formation at the cathode. While more insights into electrode–microorganism charge transfer mechanisms can be gained using impedance spectroscopy (IES), this electrochemical technique could damage electroactive biofilm due to generation of reactive species at the electrode and pH changes. Nevertheless, detailed electrochemical characterization, which also can include measuring current distribution in 3-dimensional electrodes comprising multiple c-rings, might be of interest for future studies.

Previous studies of bioelectrochemically enhanced anaerobic reactors have emphasized the substantial presence of acetoclastic microorganisms at the cathode [53,54]. In particular, significant presence of *Methanosarcinales* was observed in a bioelectrochemical reactor equipped with stainless steel electrodes [28], while in another study, hydrogenotrophic *Methanobacterium* and *Methanocorpusculum* dominated cathodic biofilm of a microbial electrolysis cell [31]. These species are capable of producing CH_4_ both from acetate and H_2_. Given the high acetoclastic activity of the inoculum sludge, acetate produced by the acetogenic bacteria is expected to be converted to CH_4_. However, considering the high acetate concentration at elevated organic loads, the proliferation of hydrogenotrophic methanogens can be considered as a more plausible explanation for increased CH_4_ production in the BEAD reactor. Indeed, a significant contribution of hydrogenotrophic methanogens to CH_4_ production in microbial electrolysis and microbial electrosynthesis cells has been previously observed [14,55,56]. Furthermore, CH_4_ production through direct electron transfer can be hypothesized [18,27,57].

Evaluating the contribution of bioelectrochemical pathways to the overall observed CH4 production involved calculating the difference between CH_4_ produced in the BEAD and control reactors and comparing it with the anticipated bioelectrochemical CH_4_ production. The latter was determined based on the assumption of 100% Coulombic Efficiency in Equation (1). According to this calculation, at an OLR of 6.6 g (L_R_ d)^−1^, only 11% of the difference between CH_4_ production in the BEAD and Control reactors (SPS-separated feedstock) can be attributed to bioelectrochemical CH_4_. This value somewhat increased in the experiment conducted at the NRC laboratory (Montreal) using leachate as a feedstock, where the highest current of 11.9 mA was observed at an OLR of 8.3 g (L_R_ d)^−1^ (Figure 3). Consequently, in this test bioelectrochemical CH_4_ production accounted for 25–30% of the increase in CH_4_ production. Notably, since these values are based on the assumed 100% Coulombic efficiency, the calculated values represent an upper limit of the bioelectrochemical contribution. The actual contribution to CH_4_ production is expected to be lower due to electron losses to competing reactions (e.g., redox processes, biomass growth).

Considering that CH_4_ production in BEAD was increased up to 30% as compared to Control reactor and exceeded the estimations based on the observed current, a significant part of this increase can be attributed to the improved interspecies electron transfer (IET) within the anodic and cathodic biofilms. Indeed, several previous studies demonstrated increased CH_4_ production in the presence of conductive materials such as carbon powder and biochar, which facilitated electron exchange between different members of the mixed anaerobic microbial consortium [58,59]. 

Detailed biomolecular characterization of biofilms and suspended microbial populations of the BEAD reactor would be instrumental in enhancing our understanding of this complex system. Additionally, such detailed characterization of microbial populations might help to understand and differentiate between contributions of microbial biofilms formed at the anode and cathode surface and suspended biomass, including granular biomass introduced to the reactor during its inoculation. The improved performance of an anaerobic digestor equipped with electrodes was previously observed and attributed to improved biomass (biofilm) retention [60].

Furthermore, several previous AD-MEC studies have reported improved CH_4_ production and yield, consistent with the results of this study. For example, Yin et al. [61] reported a 24% increase in CH_4_ yield in AD-MEC tests using synthetic wastewater, while a 22% increase was observed in an AD-MEC system fed with waste activated sludge [62]. Even greater improvements in CH_4_ production and yield have been observed in other studies. For instance, Park et al. [17] reported a 1.7-fold increase in CH_4_ production and nearly 4-fold reduction in stabilization time. Although none of these studies employed the same reactor and electrode design making direct performance comparison difficult, it can be concluded that the results of this study agree well with the broadly observed trend of improved reactor performance and stability in AD-MEC systems.

### 3.3. Implications of Using BEAD for Food Waste Conversion to Biogas

The experimental results described above provide strong evidence of enhanced biogas production under bioelectrochemical conditions. The increased CH_4_ production enables more efficient renewable energy recovery from organic waste. Importantly, in addition to the increased CH_4_ production, reactor stability also improved. The stability of anaerobic reactors is of utmost importance for successful application of AD technology, particularly in small and remote communities. Such communities often rely on high-cost fossil fuels for energy production and lack alternative sources of energy due to limited agricultural activity and harsh climate that limits solar and wind energy generation. In these communities, food waste serves as a viable source of renewable energy. Nevertheless, attempts to utilize biodegradable food waste for biogas production are hindered by the high cost of anaerobic reactors and the substantial expertise required for successful reactor operation. Consequently, a significant amount of food waste is landfilled, posing environmental challenges such as the contamination of the delicate northern environment with leachates rich in nitrogen and phosphorus. Moreover, anaerobic degradation of landfilled food waste results in a considerable release of CH_4_, a potent greenhouse gas, into the atmosphere [63].

In more temperate climates, the high sensitivity of AD to feedstock composition often results in reactors being operated at low organic loads. Many anaerobic digesters are built using the continuous stirred tank reactor (CSTR) design rather than a more efficient high-rate UASB design due to the simpler operation and higher stability of CSTR reactors. Such CSTR-based anaerobic digesters treating food waste are typically operated at OLR in the range of 2–5 g COD (L d)^−1^ to avoid excessive VFA accumulation and process instability. Operation such OLR ranges necessitate long hydraulic retention times of 20–30 days [5,6,7] and oversized reactor volumes to maintain stable performance. In contrast, the BEAD reactor evaluated in this study demonstrated stable operation and enhanced methane production at organic loading rates up to 7–8 g COD (L d)^−1^, even under conditions that led to organic overload in the control reactor. This highlights the potential of integrating bioelectrochemical pathways with high-rate reactor configurations to achieve high volumetric methane productivity without compromising process stability.

## 4. Conclusions

This proof-of-concept study evaluated CH_4_ production in a high-rate UASB-based BEAD reactor and compared its performance with that of a conventional UASB reactor. To ensure the robustness of the proposed bioelectrochemical approach, the reproducibility of observed trends, and the broad applicability of the proposed reactor design, experiments were conducted in two different laboratories using similar reactor setups but distinct feedstocks and operating temperatures. Also, to assess long-term reactor performance, both the BEAD and Control reactors were operated for a combined total of 141 days.

In all experiments, the BEAD reactor consistently demonstrated enhanced CH_4_ production and yield, as well as improved reactor stability under high organic loading conditions, compared to the Control reactor. On average, CH_4_ production and yield in the BEAD reactor were increased by 20–30%. Notably, this improvement increased to nearly 50% when the reactors were operated with SPS liquid at the highest tested organic load, which caused overloading of the Control, whereas the BEAD reactor maintained stable performance. These results suggest that bioelectrochemical systems can significantly enhance the activity of electroactive microorganisms, leading to increased methane output and greater reactor stability.

While the current experimental results provide compelling evidence of the BEAD reactor improved performance compared to the conventional UASB design, scaling up the BEAD approach requires long-term studies to validate its performance over extended periods (e.g., beyond 6 months) and at even lower temperatures. Notably, bioelectrochemical systems, such as microbial electrolysis cells, have previously demonstrated effective operation at low mesophilic temperatures, e.g., 15–25 °C. Given the substantial energy requirements for maintaining a typical mesophilic temperature of 35–37 °C in a UASB reactor, operating the BEAD reactor at lower mesophilic temperatures could significantly reduce energy consumption in temperate and cold climates facilitating BEAD technology acceptance. Moreover, a comprehensive techno-economic assessment is required to identify organic loading rates and reactor operating temperatures that balance CH_4_ production, energy input, and process stability for full-scale applications.

## Figures and Tables

**Table 1:** Experimental conditions during BEAD and Control reactor experiments. OLR values represent the average of 5–8 measurements with standard deviations estimated to be below 7% of each value.

Test #	Location	Feedstock	OLR g (L_R_ d)^−1^	HRT Day	Temperature, °C	BEAD Voltage (V)	Duration Day
1-1	Montreal	FW leachate, centrifuged	4.3	4	22	1.4	16
1-2	Montreal	FW leachate, non-centrifuged	8.3	4	22	1.4	18
1-3	Montreal	FW leachate, non-centrifuged	7.7	4	22	1.4	13
2-1	Ottawa	FW leachate, centrifuged	3	4	22	1.4	12
2-2	Ottawa	FW leachate, non-centrifuged	3	4	22	1.4	15
2-3	Ottawa	FW leachate, non-centrifuged	5	4	22	1.4	18
2-4	Ottawa	FW leachate, non-centrifuged	7	4	22	1.4	23
3-1	Montreal	FW SPS liquid	4.5	8.8	35	1.2	8
3-2	Montreal	FW SPS liquid	6.6	6.4	35	1.2	12
3-3	Montreal	FW SPS liquid	5.7	6.5	35	1.2	6

**Table 2:** FW leachate and SPS liquid characterization. Values correspond to concentrations in the reactor influent stream, i.e., after feedstock dilution. Volatile fatty acid (VFA) and COD values represent the average of 5–8 measurements with standard deviations below 5% of each value.

Test	Substrate	tCOD g L^−1^	sCOD g L^−1^	OLR (g (L_R_ d)^−1^	Acetate mg L^−1^	Propionate mg L^−1^	Butyrate mg L^−1^
1-1	Centrifuged leachate (Montreal)	n/a	18.4	4.3	6510	1150	3100
1-2	Non-centrifuged leachate (Montreal)	22.8	10.6	7.7; 8.3	3615	570	1780
1-3							
2-1	Centrifuged leachate (Ottawa)	n/a	15	3	4788	2268	5166
2-2	Non-centrifuged leachate (Ottawa)	15.1	10.3	3	3990	1890	4305
2-3	Non-centrifuged leachate (Ottawa)	24.8	17.4	5	6597	2951	7991
2-4	Non-centrifuged leachate (Ottawa)	35.2	24.5	7	9310	4165	10,291
3-1, 3-2, 3-3	SPS liquid (Montreal)	n/a	16.3	4.5–6.6	1490	648	1530

## Data Availability

The original contributions presented in this study are included in the article. Further inquiries can be directed to the corresponding authors.
